# Refining microbial biomarker identification in rumen microbiome studies: a viability PCR-based approach

**DOI:** 10.1128/aem.01429-25

**Published:** 2025-09-19

**Authors:** Woohyung Lee, Geonwoo Kim, Tansol Park

**Affiliations:** 1 Department of Animal Science and Technology, Chung-Ang University 65479 , Anseong-si, Gyeonggi-do, South Korea; Universidad de los Andes, Bogotá, Colombia

**Keywords:** viability polymerase chain reaction, propidium monoazide, rumen microbiome, biomarker

## Abstract

The rumen microbiome significantly affects host performance, influencing feed efficiency, nitrogen utilization, and methane emission. However, conventional DNA-based marker gene sequencing cannot distinguish between viable and non-viable microbes, leading to inaccurate microbiota analyses. Viability PCR (v-PCR) with propidium monoazide (PMA) can inhibit DNA amplification from membrane-compromised cells, allowing the detection of viable microbes in rumen cultures. Therefore, this study aims to identify conditions for applying PMA in rumen culture experiments using qPCR and to examine its effect on the rumen microbial community using 16S rRNA gene sequencing in standard *in vitro* experiments. PMA treatment conditions were applied using a fivefold inoculum dilution, 100  µM PMA concentration, 30 min dark incubation, and 20 min light exposure, validated by a decrease in absolute abundance in heat-treated samples. When applied to *in vitro* rumen experiments, PMA treatment reduced bacterial evenness and induced shifts in key bacterial and archaeal taxa. Additionally, it affected major functional profiles of the microbiota. PMA treatment increased the relative abundance of *Ruminobacter* [log fold change (LFC) = 0.52] and *Succinivibrio* (LFC = 0.68) at 0 h (no incubation), along with *Ruminobacter* (LFC = 0.83) after 24 h of incubation, while decreasing that of *Xylanibacter* (LFC = −0.39) at 24 h. These shifts align with those of RNA-based studies showing higher Succinivibrionaceae abundance than Prevotellaceae, supporting the effectiveness of PMA in capturing active microbial dynamics. PMA-based v-PCR offers a reliable alternative to RNA-based methods, improving microbial community assessments and facilitating the identification of viability-associated microbial biomarkers in rumen studies.

**IMPORTANCE:**

This study identifies the optimal conditions for applying propidium monoazide (PMA) in *in vitro* rumen experiments to selectively amplify DNA from viable microorganisms while suppressing amplification from nonviable ones. PMA-based viability PCR (v-PCR) improves the accuracy of microbial community analysis by selectively detecting viable microorganisms, addressing the limitations of conventional DNA-based methods. Additionally, this approach provides a potential cost-effective alternative to RNA-based analyses, offering a practical tool for studying rumen microbial ecology.

## INTRODUCTION

A comprehensive understanding of the rumen microbiome is crucial for addressing key challenges in ruminants, including methane mitigation, improved nitrogen utilization efficiency, and increased productivity. This microbiome provides insights into the microbial drivers and agents modulating feed digestion and fermentation. While traditional DNA-based microbiome analyses identify potential microbial residents in the rumen ecosystem, RNA-based approaches provide direct evidence of microbial activity, offering a more accurate representation of the active microbiome. The discrepancy between total and viable cell counts in rumen fluid can be as much as 10-fold (1). Similarly, in fecal samples compositionally comparable to rumen samples, nonviable cells constitute approximately 32% of the microbial population (2). Considering DNA remains amplifiable for several days post-cell death (3), DNA-based microbial community analysis may overestimate microbial abundance and misrepresent actual functional activity (4). Messenger RNA (mRNA) serves as a reliable indicator of living cells due to its exclusive synthesis in active cells (5, 6). Additionally, RNA from dead cells degrades more rapidly than DNA (7, 8), making RNA-based analysis a potentially more accurate alternative to DNA-based methods. However, in slow-growing cells, RNA content may fall below the polymerase chain reaction (PCR) detection threshold despite cellular viability and activity (9). Also, preserving intact RNA presents significant technical challenges. Given that RNA is detected only in metabolically active microorganisms at the time of sampling, its recovery is more time-sensitive than DNA (10). The efficiency of RNA extraction varies by methods, with studies indicating that gram-negative bacteria typically yield higher RNA extraction rates than gram-positive bacteria (11).

To address these limitations, viability PCR (v-PCR) was introduced using ethidium monoazide (EMA) in 2003 (12). EMA inhibits DNA amplification from bacterial cells with compromised membranes (13). However, EMA can inhibit DNA amplification in viable bacteria (14–17), limiting its specificity. Propidium monoazide (PMA), an alternative with two positive charges compared to the single charge of EMA (18), is less likely to penetrate membrane-intact cells. This property makes PMA more effective in selectively amplifying DNA from viable microorganisms, enabling a more accurate analysis of active microbial communities.

Both EMA and PMA have been applied to various microorganisms—including bacteria (19–25), fungi (26), viruses (27, 28), protozoa (29, 30), yeast (31), and archaea (32)—along with diverse environmental samples, such as marine environments (33), water (19, 21, 34, 35), feces (36–39), soil (20, 40), and sludge (41). However, PMA has not been applied to the rumen microbiome, and its implementation presents challenges due to the high turbidity caused by the dense microbial population reaching approximately 10^10^ cells per mL of rumen fluid (42). This turbidity is further exacerbated by a large number of feed particles and dead cells (1), reducing the light intensity necessary for photoactivation and limiting dye molecule availability per cell, potentially compromising v-PCR efficiency (13). To mitigate these issues, reducing sample turbidity is essential.

Attempts to increase PMA concentrations in particle-rich samples (e.g., from 50 to 130 µM) have failed to inhibit the amplification of dead bacterial DNA (32). Studies indicate that turbidity levels greater than 10 Nephelometric Turbidity Units (NTU) interfere with PMA treatment, while values below this threshold show no interference (21, 24). Dilution has been suggested as a strategy to reduce turbidity (32), with one study reporting that a 100-fold fecal sample dilution was the most effective (39). In other studies, 45-fold and 10-fold dilutions have been used for similar purposes (37, 38). To effectively apply PMA in rumen samples, the appropriate dilution ratio must be determined to minimize the amplification of DNA from nonviable cells.

Therefore, this study aimed to evaluate how sample dilution affects the selective amplification of viable microbial DNA in *in vitro* rumen experiments and to apply PMA-based v-PCR accordingly. Rumen fluid was mixed with *in vitro* buffer at a 1:2 ratio. Subsequently, the inoculum was tested undiluted, as well as at fivefold and tenfold dilutions, to evaluate v-PCR performance and verify PMA efficacy. The PMA application protocol, comprising PMA concentration, incubation time, and light exposure duration, was informed by fecal sample studies with composition similar to that of the rumen fluid (37–39). We hypothesized that substrate depletion during incubation would result in microbial death, thereby revealing differences between conventional DNA analysis and PMA-treated analysis. To test this hypothesis, incubation experiments were conducted at 0, 24, and 48  h, followed by application of the PMA treatment protocol to determine the viable microbial contribution at each time point.

## RESULTS

### PMA treatment effects in heat-treated and non-treated groups (Experiment 1)

**Fig 1:**
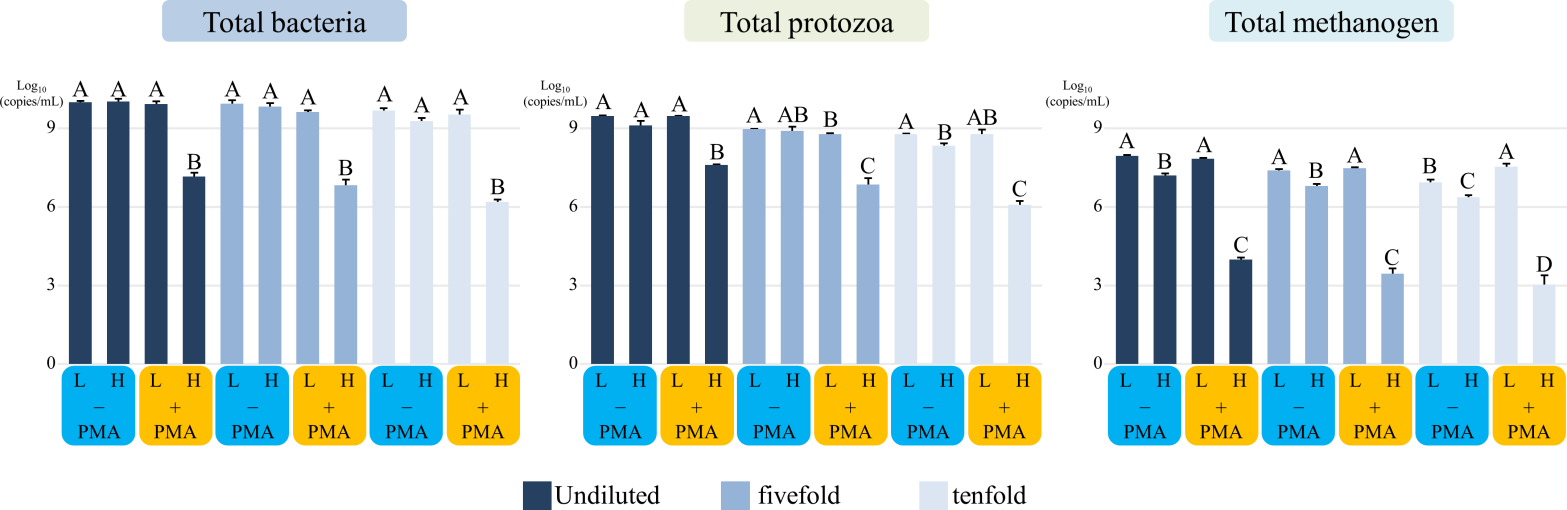
Absolute abundance of rumen microbial guilds to evaluate the effect of PMA treatment (+ PMA) under heat-treated and non-treated conditions (Experiment 1). A–D, means (*n* = 3) within each dilution group followed by different superscript letters indicate significant differences between PMA-treated and untreated samples (*P* < 0.05).Statistical comparisons were performed separately within each dilution level (undiluted, fivefold, and tenfold) for each microbial group (total bacteria, protozoa, and methanogens). L, non-heat-treated groups; H, heat-treated groups; − PMA, non-PMA-treated samples; + PMA, PMA-treated samples. Values represent the means of three technical replicates derived from pooled rumen fluid collected from five Hanwoo cows.

To assess changes in the absolute abundance of rumen microbial guilds after PMA treatment, quantitative PCR (qPCR) targeting total bacteria (TB), total protozoa (TP), and total methanogens (TM) was conducted. The absolute abundance changes induced by PMA treatment in heat-treated and untreated groups are summarized (Fig. 1). For each microbial target, statistical comparisons were conducted within each dilution group (no dilution, fivefold, and tenfold). In all dilution conditions, heat-treated samples followed by PMA treatment consistently showed the lowest absolute abundance (*P* < 0.05). In contrast, in non-heat-treated samples, PMA treatment reduced protozoal abundance in the fivefold dilution group, while methanogen abundance increased in the tenfold dilution group (*P* < 0.05).

### *In vitro* fermentation parameters according to incubation times (24 h, 48 h; Experiment 2)

To validate the biological relevance of the *in vitro* system for PMA-based microbial analysis, fermentation parameters were measured after 24 and 48 h of incubation (Table S1). Extending the incubation time from 24 h to 48 h significantly decreased pH and acetate (%) (*P* < 0.05). In contrast, no significant differences were observed in propionate (%), butyrate (%), and the acetate-to-propionate (A:P) ratio. Other fermentation parameters (DMD [%], NDFD [%], ADFD [%], NH_3_-N [mg/dL], total gas [mL], CH_4_ [mL], CH_4_ [mL/g dDM], total VFA [mM], BCVFA [%], iso-butyrate [%], and iso-valerate [%]) showed a significant increase as incubation time progressed (*P* < 0.05).

### Quantitative analysis of microbial guilds according to PMA treatment and incubation times (0 h, 24 h, and 48 h; Experiment 2)

The effects of PMA treatment and incubation time on the absolute abundances of rumen microbial guilds were evaluated using qPCR. PMA treatment significantly reduced the absolute abundances of TB, total fungi (TF), and *Methanobrevibacter* spp. (MBB) (*P* < 0.05) (Table 1). The absolute abundances of TB and TP significantly decreased at 48 h compared to 0 h and 24 h (*P* < 0.001), while *Entodinium* spp. (ENT) showed a continuous decline over time. In contrast, TM, TF, MBB, and *Methanomicrobium* spp. (MMB) exhibited varying patterns: TM was lowest at 0 h, TF and MMB peaked at 48 h, and MBB increased consistently over time. The abundance of the methyl coenzyme M reductase gene (*mcrA*) increased significantly at 24 h but subsequently declined at 48 h (*P* < 0.01). Significant interactions between PMA treatment and incubation time were observed for TB, TM, ENT, MBB, and *mcrA* (*P* < 0.05).

**TABLE 1:** Absolute abundance (log_10_ copies/mL) of rumen microbial guilds according to PMA treatment and incubation time (Experiment 2)^*a*^

Microbial guilds^*b*^	Treatment		Time			Pooled SEM^*c*^	*P*-value		
	− PMA	+ PMA	0 h	24 h	48 h		Treatment	Time	T × T^*d*^
TB	10.51^A^	10.21^B^	10.43^A^	10.42^A^	10.22^B^	0.10	<0.0001	0.0004	<0.0001
TP	9.69	9.68	9.99^A^	9.90^A^	9.17^B^	0.15	0.5024	<0.0001	0.2928
TM	7.75	7.72	7.61^B^	7.75^A^	7.84^A^	0.13	0.6124	0.0010	0.0107
TF	5.74^A^	5.41^B^	5.43^B^	5.36^B^	5.94^A^	0.39	0.0097	0.0096	0.8405
ENT	7.47	7.40	8.10^A^	7.89^B^	6.32^C^	0.19	0.0907	<0.0001	0.0259
DAS	6.34	6.42	6.32	6.45	6.37	0.21	0.4143	0.3716	0.4989
MBB	7.90^A^	7.86^B^	7.71^C^	7.80^B^	8.13^A^	0.09	0.0427	<0.0001	0.0268
MMB	5.24	5.32	5.09^B^	5.18^B^	5.58^A^	0.14	0.1486	<0.0001	0.5731
*mcrA*	10.25	10.21	10.12^C^	10.31^A^	10.26^B^	0.04	0.0653	<0.0001	<0.0001

^*a*^ Values represent means (*n* = 5) for each PMA × time combination. Superscript letters (A–C) within treatment (− PMA; + PMA) or fermentation time (0, 24, and 48 h) columns indicate significant differences between levels of each factor, based on two-way ANOVA and Tukey’s HSD test (*P* < 0.05). Fixed factors were PMA treatment (− PMA; + PMA) and fermentation time (0, 24, and 48 h).

^*b*^ TB, total bacteria; TP, total protozoa; TM, total methanogen; TF, total fungi; ENT, *Entodinium* spp.; DAS, *Dasytricha ruminantium*; MBB, *Methanobrevibacter* spp.; MMB, *Methanomicrobium* spp.; *mcrA*, methyl coenzyme-M reductase gene.

^*c*^ Pooled SEM is presented for each microbial guild across all combinations.

^*d*^ T × T, treatment by time interaction.

Microbial guilds exhibiting significant differences in response to PMA treatment at each incubation time, based on the interaction effect (PMA × time), are summarized (Table 2). PMA treatment reduced TB abundance at 0 h and 24 h, as well as TB, TM, ENT, MBB, and *mcrA* abundance at 48 h (*P* < 0.05).

**TABLE 2:** Absolute abundance (log_10_ copies/mL) of rumen microbial guilds for the confirmation of the effects of PMA treatment at each incubation time (Experiment 2)^*a*^

Group	Absolute abundance, log copies/mL						
	0 h TB	24 h TB	48 h TB	48 h TM	48 h ENT	48 h MBB	48 h *mcrA*
− PMA	10.52^A^	10.52^A^	10.48^A^	7.96^A^	6.48^A^	8.20^A^	10.33^A^
+ PMA	10.35^B^	10.33^B^	9.95^B^	7.72^B^	6.16^B^	8.06^B^	10.19^B^
Pooled SEM	0.04	0.03	0.09	0.07	0.12	0.04	0.04
*P*-value	0.0011	0.0011	0.0011	0.0011	0.0163	0.0163	0.0011

^*a*^ Values are presented as means (*n* = 5) for each treatment group. Superscript letters (A–B) within each row indicate significant differences between PMA-treated and untreated groups (*P* < 0.05). Data were reorganized from Table 1, showing only the groups with significant differences after PMA treatment for each incubation time. This table includes only the microbial groups with significant PMA treatment effects having significant interaction effects on increasing incubation time. Groups without significant PMA treatment effects are not presented in this table. − PMA, non-treated group; + PMA, PMA-treated group; h, incubation time; 0 h, no incubation; 24 h, 24 hours of incubation; 48 h, 48 hours of incubation; TB, total bacteria; TM, total methanogen; ENT, *Entodinium* spp.; MBB, *Methanobrevibacter* spp.; *mcrA*, methyl coenzyme-M reductase gene.

### Alpha and beta diversity analyses of the ruminal microbiota (Experiment 2)

Based on 16S rRNA gene amplicon sequencing data, PMA treatment significantly decreased evenness, Shannon’s index, and Simpson’s index in bacteriota (*P* < 0.05) (Table 3). Significant differences in evenness and Simpson’s indices were observed among incubation times (*P* < 0.05). The evenness index was highest at 0 h and lowest at 24 h, while the 48 h group showed no significant difference from either. The Simpson’s index was highest at 48 h and lowest at 24 h, while the 0 h group showed no significant difference from the others. Significant interaction effects between PMA treatment and incubation time were identified only for evenness and Simpson’s indices (*P* < 0.05).

**TABLE 3:** Effects of PMA treatment and fermentation time on alpha diversity indices for ruminal microbiota (Experiment 2)^*a*^

Taxonomic group	Parameter	Treatment		Time			Pooled SEM^*b*^	*P*-value		
		− PMA	+ PMA	0h	24h	48h		Treatment	Time	T × T^*c*^
Bacteriota	Observed ASVs	1,719	1,679	1,731	1,672	1,694	235.88	0.7219	0.9435	0.4748
	Chao1	1,732	1,692	1,743	1,682	1,710	236.37	0.7219	0.9611	0.4748
	Evenness	0.902^A^	0.890^B^	0.899^A^	0.891^B^	0.897^AB^	0.01	<0.0001	0.0128	0.0288
	Faith’s phylogenetic diversity	123.90	117.47	122.50	122.99	116.57	11.95	0.2464	0.3719	0.4500
	Shannon	9.68^A^	9.52^B^	9.66	9.54	9.62	0.17	0.0199	0.4868	0.1182
	Simpson	0.9970^A^	0.9960^B^	0.9968^AB^	0.9965^B^	0.9971^A^	0.0005	0.0002	0.0324	0.0097
Archaeota	Observed ASVs	9^B^	11^A^	8^C^	9^B^	12^A^	1.86	0.0023	<0.0001	0.5133
	Chao1	9^B^	11^A^	8^C^	9^B^	13^A^	2.14	0.0024	<0.0001	0.4940
	Evenness	0.911	0.900	0.916	0.899	0.903	0.03	0.1749	0.2747	0.3094
	Faith’s phylogenetic diversity	0.95	1.00	0.90^B^	1.00^AB^	1.04^A^	0.12	0.1022	0.0100	0.2222
	Shannon	2.79^B^	3.05^A^	2.66^B^	2.86^B^	3.24^A^	0.29	0.0207	<0.0001	0.8902
	Simpson	0.830	0.854	0.818^B^	0.833^B^	0.875^A^	0.03	0.0582	0.0006	0.7162

^*a*^ Values represent means (*n* = 5) for each level of PMA treatment (− PMA; + PMA) and fermentation time (0, 24, and 48 h). Superscript letters (A–C) within treatment (− PMA; + PMA) or fermentation time (0, 24, and 48 h) columns indicate significant differences between levels within each factor, based on two-way ANOVA followed by Tukey’s HSD test (*P* < 0.05). Fixed factors were PMA treatment and fermentation time. − PMA, non-PMA-treated group; + PMA, PMA-treated group.

^*b*^ Pooled SEM is presented for each diversity index. For bacteriota, Good’s coverage exceeded 99.74%, and for archaeota, it exceeded 96.42%.

^*c*^ T × T, treatment-by-time interaction.

In archaeota, PMA treatment significantly increased the observed ASVs, Chao1, and Shannon indices (*P* < 0.05) (Table 3). Additionally, all indices except evenness varied significantly among incubation times (*P* < 0.05), with some changing only at specific time points. No interaction effect between PMA treatment and incubation time was observed.

**Fig 2:**
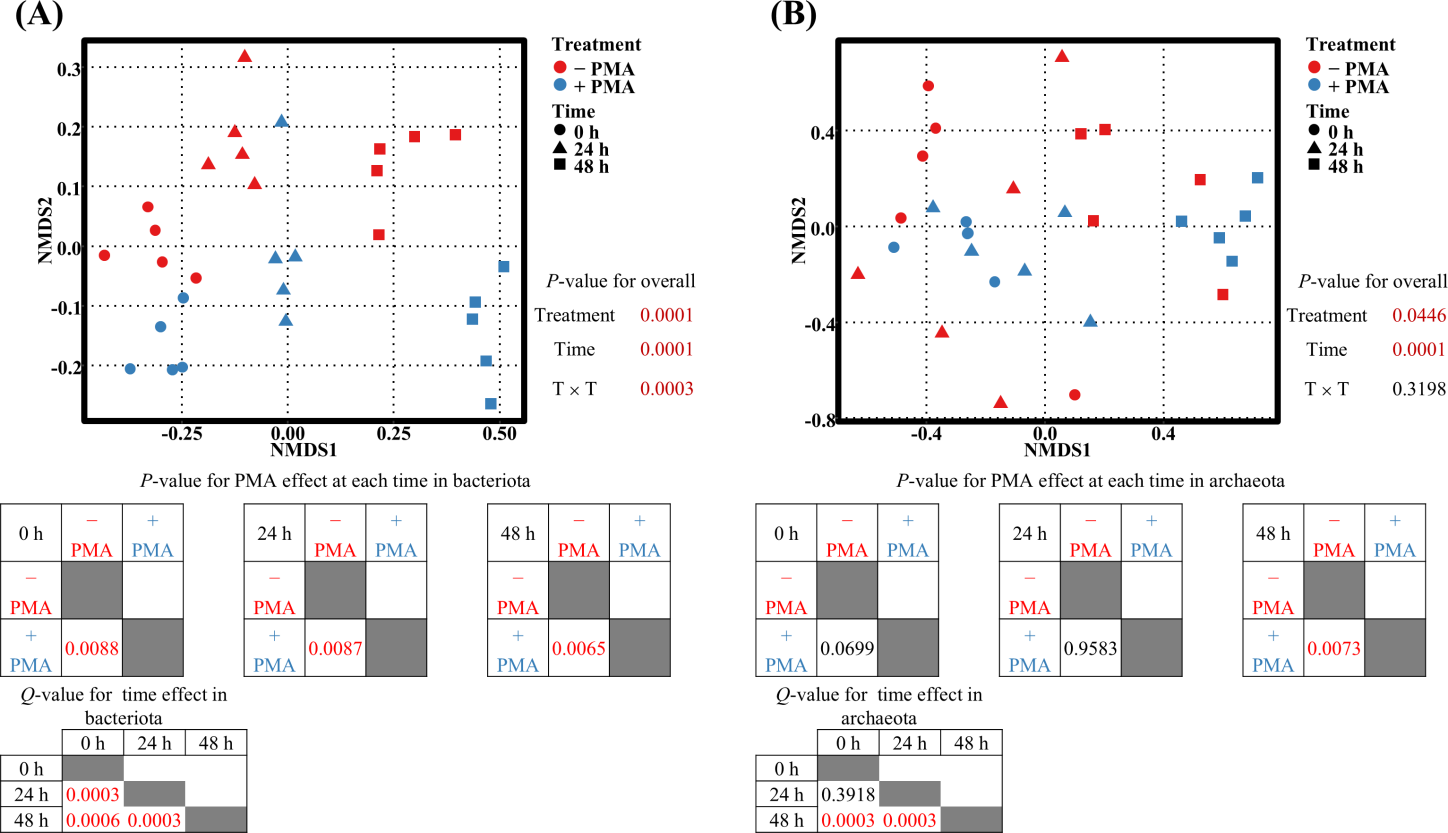
NMDS plots for ruminal bacteriota (**A**) and archaeota (**B**) based on the Bray–Curtis dissimilarity. PERMANOVA was used to analyze the effect of PMA treatment and incubation time points. − PMA, non-PMA-treated samples; + PMA, PMA-treated samples; 0 h, no incubation; 24 h, 24 hour of incubation; 48 h, 48 hour of incubation; T × T, treatment by time interaction. Each point represents a technical replicate derived from the pooled rumen fluid.

Beta diversity analysis based on Bray–Curtis dissimilarity revealed that the bacteriota community composition significantly differed by PMA treatment and incubation time (*P* < 0.001) (Fig. 2A). Pairwise comparisons showed that PMA-treated and nontreated samples were significantly separated at all incubation time points (*P* < 0.01). Additionally, significant differences among the three incubation time points were identified (adjusted using the Bonferroni-corrected *P*-value, *Q* < 0.01).

Archaeota community composition also showed significant differences according to PMA treatment and incubation time (*P* < 0.05) (Fig. 2B). In contrast, for archaeota, a significant separation between PMA-treated and nontreated groups was only observed at 48 h (*P* < 0.01). Furthermore, significant differences were observed only for the 48 h group compared to those of the other groups (*Q* < 0.01).

### Differences in microbial taxa abundance according to PMA treatment at each incubation time (Experiment 2)

**Fig 3:**
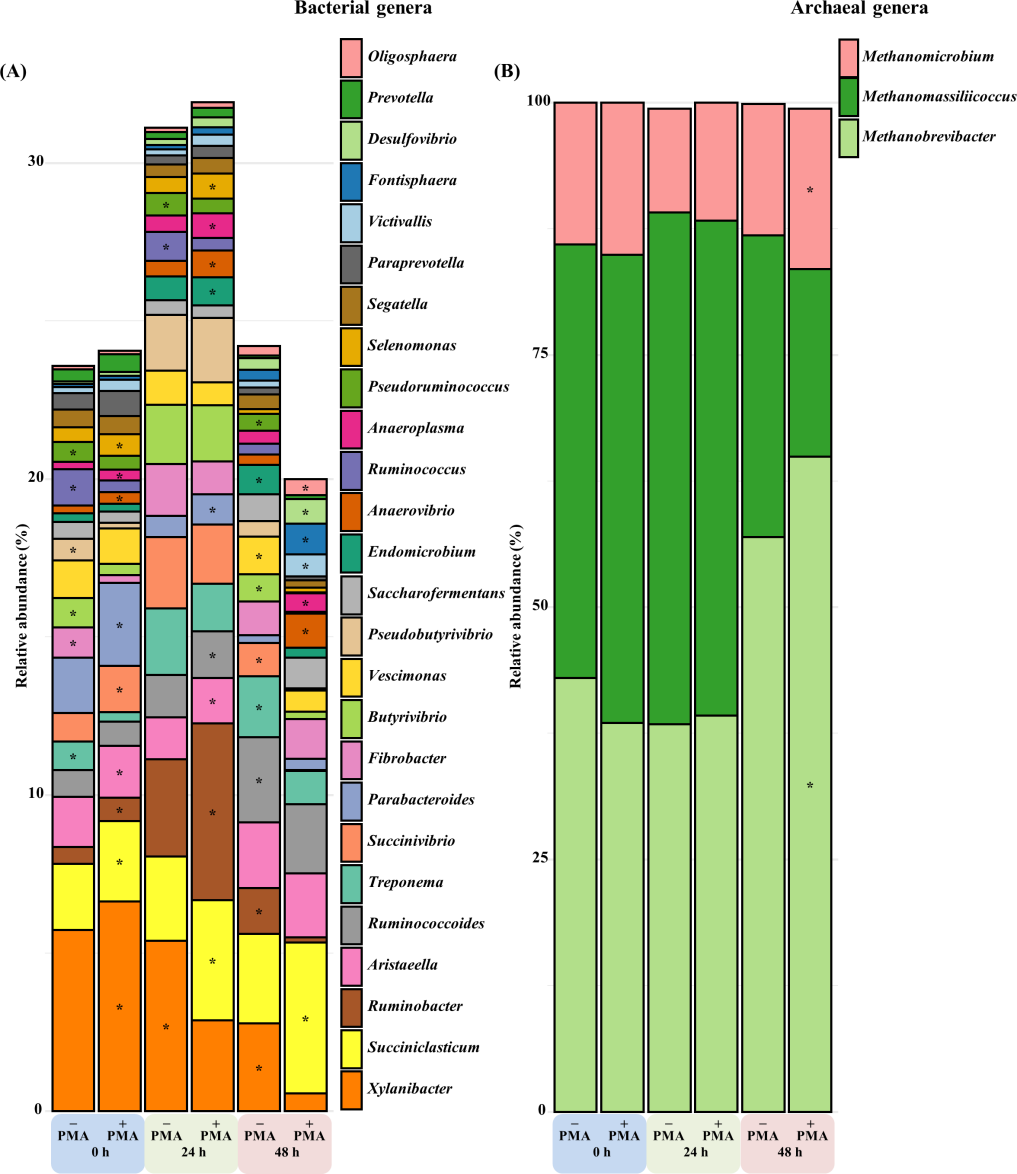
Mean relative abundance of major bacterial genera (**A**) and archaea genera (**B**) across analyzed samples. − PMA, non-PMA-treated samples; + PMA, PMA-treated samples; 0 h, no incubation; 24 h, 24 hour of incubation; 48 h, 48 hour of incubation. Taxa detected in over 60% of the samples in at least one treatment group with an average relative abundance exceeding 0.5% and classified to the genus level were visualized as bar plots of major taxa. Significant differences due to PMA treatment within each incubation time point (*Q* < 0.05) were marked, using an absolute Log-Fold Change (LFC) threshold of >0.1. Table S2 and Table S3 present LFC values of those significantly affected genera. *: Indicates significantly higher differential abundance (*Q* < 0.05) in either the − PMA or + PMA group at each time point.

For bacteriota, PMA treatment significantly altered the differential abundance of five phyla and 15 genera at 0 h, six phyla and 12 genera at 24 h, and eight phyla and 16 genera at 48 h (*Q* < 0.05) (Table 4; Table S2; Fig. 3A). At the phylum level, the differential abundance of Pseudomonadota increased at 0 h (LFC = 0.35) (*Q* < 0.01) and 24 h (LFC = 0.29) (*Q* < 0.01) but decreased at 48 h (LFC = −2.30) (*Q* < 0.05). The differential abundance of Mycoplasmatota increased throughout the incubation period (LFC = 0.52 at 0 h, 0.42 at 24 h, and 0.40 at 48 h) (*Q* < 0.01). Meanwhile, the differential abundance of Bacillota decreased at 48 h (LFC = −0.16) following PMA treatment (*Q* < 0.01). At the genus level, PMA treatment significantly reduced the differential abundance of *Succinivibrio* (LFC = −3.26) (*Q* < 0.01), *Pseudoruminococcus* (LFC = −2.97) (*Q* < 0.01), *Ruminobacter* (LFC = −2.54) (*Q* < 0.01), and *Xylanibacter* (LFC = −1.66) (*Q* < 0.01) at 48 h (Table S2).

**TABLE 4:** Differential abundance analysis of bacterial and archaeal phyla to identify the effects of PMA treatment at each incubation time point (Experiment 2)^*a*^

Taxonomic group	Incubation time	Phylum	Log-fold change	*Q-*value
Bacteriota	0 h	Mycoplasmatota	0.52	<0.001
		Pseudomonadota	0.35	0.003
		Planctomycetota	0.23	0.024
		Spirochaetota	−0.63	<0.001
		Fibrobacterota	−1.28	<0.001
	24 h	Lentisphaerota	0.51	<0.001
		Mycoplasmatota	0.42	<0.001
		Planctomycetota	0.35	<0.001
		Pseudomonadota	0.29	<0.001
		Elusimicrobiota	0.18	0.008
		Fibrobacterota	−0.41	0.023
	48 h	Lentisphaerota	0.75	<0.001
		Verrucomicrobiota	0.73	<0.001
		Thermodesulfobacteriota	0.68	<0.001
		Mycoplasmatota	0.40	<0.001
		Bacillota	−0.16	0.002
		Spirochaetota	−0.34	<0.001
		Elusimicrobiota	−0.95	<0.001
		Pseudomonadota	−2.3	<0.001
Archaeota	0 h	Candidatus Thermoplasmatota	0.14	0.001
	48 h	Euryarchaeota	0.32	<0.001
		Candidatus Thermoplasmatota	−0.28	<0.001

^*a*^ The table includes only major bacterial and archaeal phylum, found in more than 60% of the samples in at least one treatment group with an average relative abundance of at least over 0.5% were presented, which significantly affected by PMA treatment (*Q* < 0.05), with an absolute Log-Fold Change (LFC) threshold of >0.1. Positive LFC values indicate higher abundance in the PMA treatment group, while negative LFC values indicate higher abundance in the DNA group. No significant PMA treatment effects were observed at 24 h of incubation for archaeota.

For archaea, PMA treatment increased the abundance of Candidatus Thermoplasmatota at 0 h (LFC = 0.14) (*Q* < 0.01) (Table 4). At 48 h, Euryarchaeota increased (LFC = 0.32) (*Q* < 0.01), while Candidatus Thermoplasmatota decreased (LFC = −0.28) (*Q* < 0.01). At the genus level, significant changes were observed only in *Methanobrevibacter* (LFC = 0.62) (*Q* < 0.01) and *Methanomicrobium* (LFC = 0.68) (*Q* < 0.01) at 48 h (Table S3; Fig. 3B).

### Effects of PMA treatment on the profiles of predicted microbial functions of ruminal microbiota at each incubation time point (Experiment 2)

**Fig 4:**
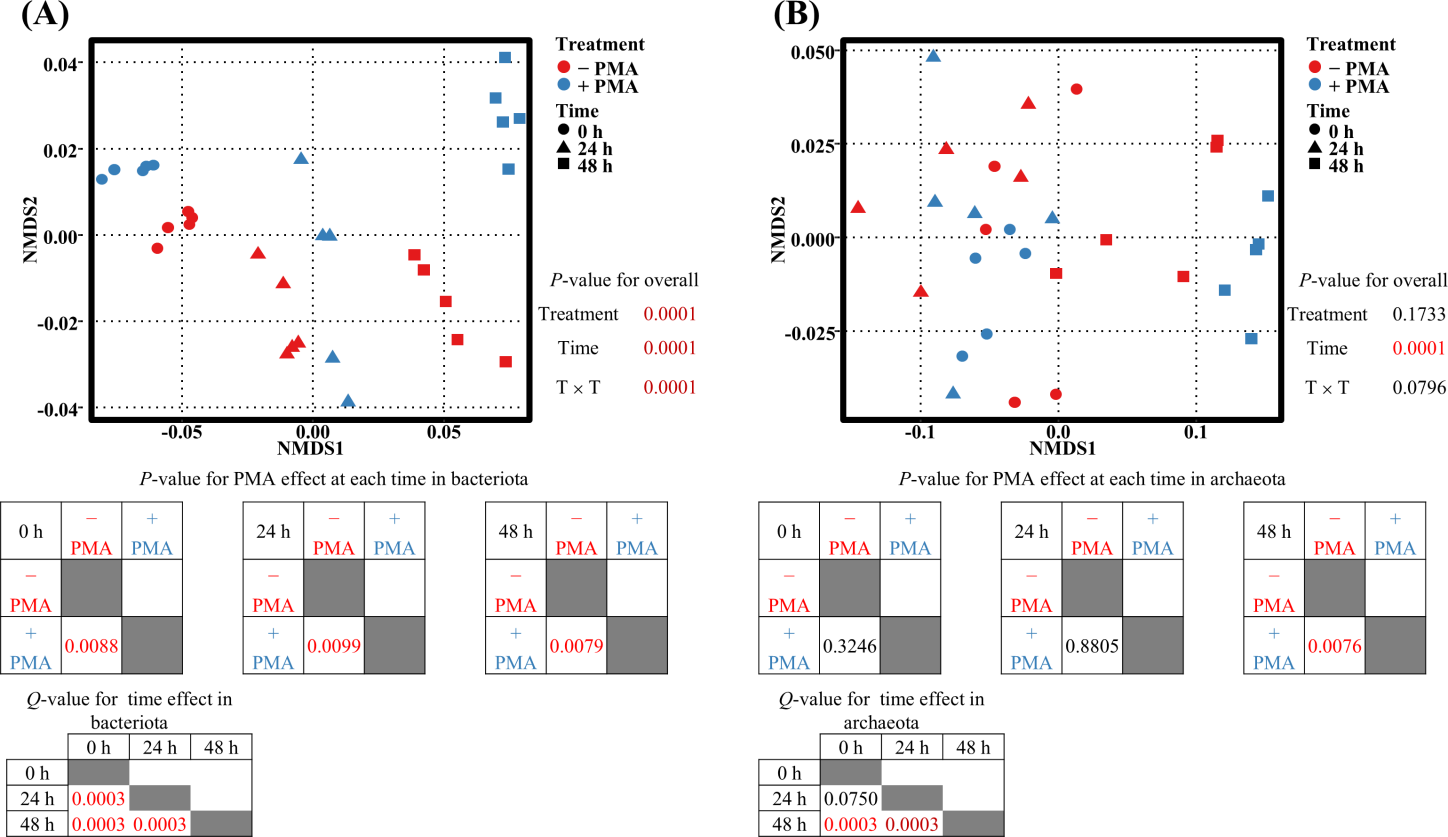
NMDS plots for the bacterial functional profile (**A**) and archaeal functional profile (**B**) based on Bray-Curtis dissimilarity of predicted EC abundance data. PERMANOVA was used to analyze the effects of PMA treatment and incubation times. − PMA, non-PMA-treated samples; + PMA, PMA-treated samples; 0, no incubation; 24 h, 24 hour of incubation; 48 h, 48 hour of incubation; T × T, treatment by time interaction. Each point represents a technical replicate derived from pooled rumen fluid.

PERMANOVA revealed that PMA treatment and incubation time significantly influenced the bacterial functional profiles based on EC abundance data (*P* < 0.01). Significant differences were observed between PMA-treated and untreated groups at each time point, as well as among all incubation time groups (*P* < 0.01, *Q* < 0.01) (Fig. 4A).

For the archaeal functional profiles, the incubation time significantly influenced the result, while PMA treatment had no significant effect (*P* < 0.01). PMA effects emerged only at 48 h, with significant differences observed exclusively between the 48 h group and other incubation time points (*P* < 0.01, *Q* < 0.01) (Fig. 4B).

### Differences in predicted functional abundances according to PMA treatment at each incubation time (Experiment 2)

**Fig 5:**
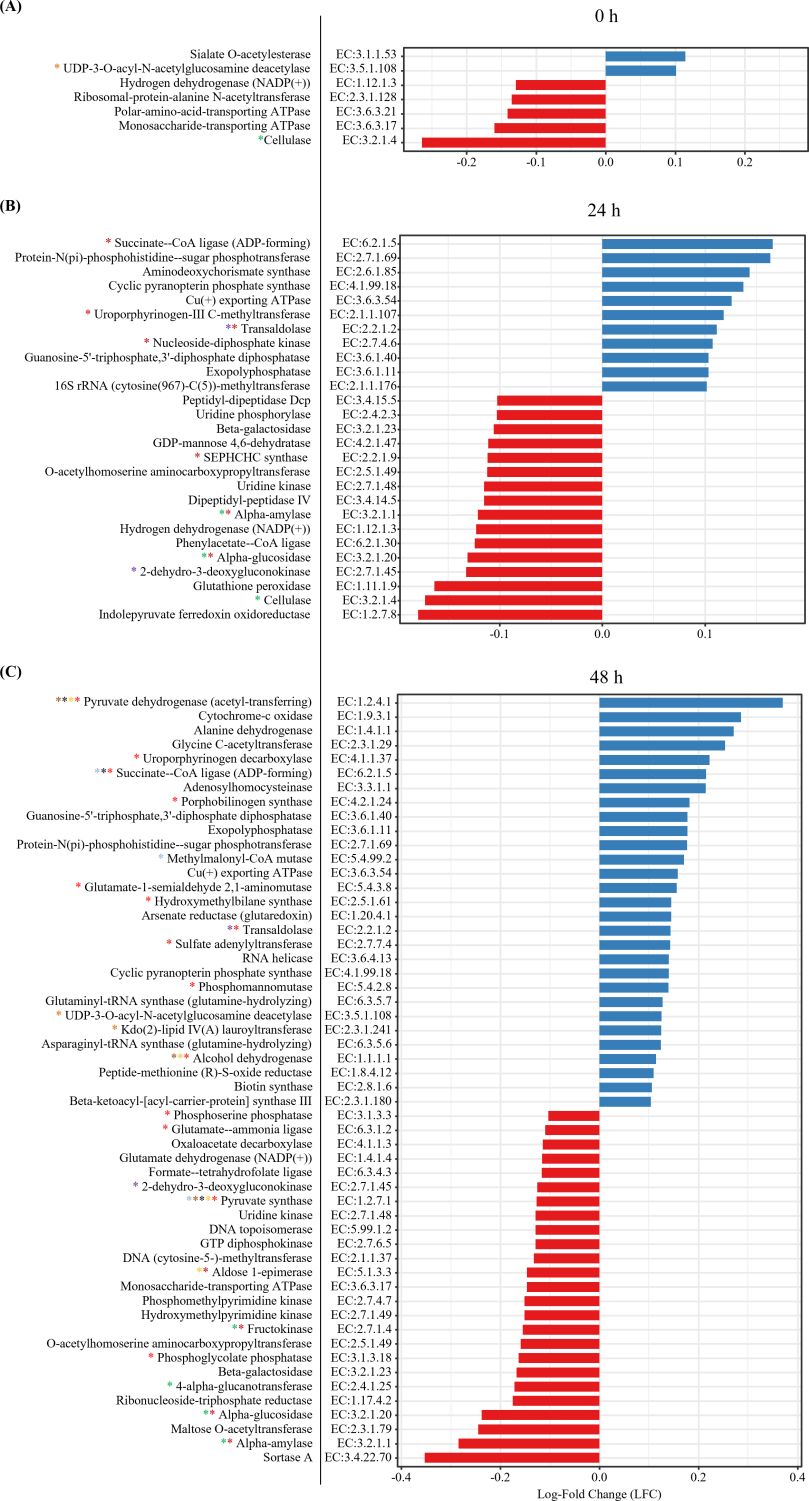
Differential abundance analysis of predicted bacterial EC abundance to assess the effect of PMA at 0 h (**A**), 24 h (**B**), and 48 h (**C**) of incubation. Significantly different ECs (*Q* ≤ 0.05) present in over 60% of samples in at least one treatment group with an average relative abundance exceeding 0.1% are shown. Positive LFC (blue color) indicates increased abundance in the PMA-treated groups, while negative values (red color) indicate decreased abundance. The color coding for asterisks is as follows: red asterisk, associated with biosynthesis of secondary metabolites; brown asterisk, associated with pyruvate metabolism; black asterisk, associated with citrate cycle (TCA cycle); yellow asterisk, associated with glycolysis and gluconeogenesis; green asterisk, associated with starch and sucrose metabolism; light blue asterisk, associated with propanoate metabolism; purple asterisk, associated with pentose phosphate pathway; orange asterisk, associated with lipopolysaccharide biosynthesis,

To analyze the effects of PMA treatment, ANCOM-BC was used to analyze differential abundance in EC abundance at each incubation time point. PMA treatment significantly altered bacterial functions, with changes in the EC abundance observed at 0 h (seven changes), 24 h (27 changes), and 48 h (54 changes) (*Q* < 0.05) (Fig. 5). The differences at 24 h became slightly more pronounced at 48 h, including pathways related to starch and sucrose metabolism and the pentose phosphate pathway. Additionally, at 48 h, differences in EC abundance were observed in glycolysis and gluconeogenesis, propanoate metabolism, pyruvate metabolism, citrate cycle, lipopolysaccharide biosynthesis, and others.

At 48 h, PMA treatment resulted in significant alterations in predicted archaeal functions, with 21 ECs showing differential abundance related to methane metabolism, pyruvate metabolism, and other associated pathways (*Q* < 0.05) (Fig. S1).

## DISCUSSION

### Application of PMA to suppress DNA signals from heat-damaged rumen microbes

The application of PMA in *in vitro* experiments to simulate ruminal fermentation and its effect on microbial analyses was investigated in this study. Based on microbial absolute abundance measurements, a fivefold inoculum dilution was selected for PMA treatment as it effectively reduced the amplification of DNA from heat-damaged ruminal microbes and protozoa presumed to be nonviable, when combined with 100  µM of PMA, 30 min dark incubation, and 20 min light exposure. Excessive dilution minimized discrepancies in DNA amplification between PMA-treated and untreated samples, thereby reducing PMA efficiency and increasing the risk of misleading results. A similar effect was reported by Papanicolas et al. (39), where over-dilution decreased DNA amplification near detection thresholds, irrespective of PMA treatment.

### Difference in absolute abundance of microbial guilds by PMA treatment and incubation time in relation to *in vitro* fermentation parameters

After applying the PMA protocol, we examined its effect on microbial analysis across different incubation time points (0, 24, and 48 h). To better assess the effects of PMA treatment, we extended the incubation time from 24 h to 48 h. Previous studies suggest that rumen fermentation experiments using a 48 h incubation period can successfully mimic the rumen microbiome, including microbial composition and VFA profiles (43). PMA treatment affected alpha diversity, shifted overall microbial and functional community structures, and altered the abundance of microbial taxa and their associated functional features. This suggests that a fraction of the microbial community may have lost viability during extended *in vitro* fermentation, possibly due to substrate depletion and were subsequently excluded by PMA treatment targeting membrane-compromised cells.

Incubation time affected various measurements from the rumen inoculum, including the microbial analysis results. The absolute abundance of TB, TP, and ENT decreased at 48 h compared to that at 0 h. Conversely, the absolute abundance of TM, TF, MBB, and MMB increased. These findings align with those of previous studies comparing 24 h and 72 h incubations (44). Here, fermentation data collected after 24 h and 48 h of incubation showed that doubling the incubation time increased DMD by approximately 9%, DNFD by 32%, and ADFD by 83%. These findings suggest that substrate depletion may have reduced the absolute abundance of certain bacteria and protozoa. Among methanogens, approximately 78% were hydrogenotrophic, utilizing H_2_ and CO_2_ to produce methane. The remaining 22% were methylotrophic, metabolizing compounds such as methanol (45). The increase in methanogen abundance at 48 h likely indicates their ability to utilize H_2_ produced by other microbes (46). Fungi exhibit a superior ability to degrade plant cell walls than that of bacteria (47), with *Neocallimastix* sp. strain releasing approximately 95% of fermentable sugars from plant material within a 4-day culture (48). This suggests that fungi can outcompete bacteria for complex substrates and survive on those that bacteria struggle to utilize for up to 48 h. Furthermore, H_2_ produced by fungi supports methanogen activity, forming a mutualistic relationship (49). This interaction was further confirmed by a co-culture experiment showing decreased fungal cellulose degradation when methanogen activity was suppressed (50). To account for the significant interaction between incubation time and PMA treatment, we analyzed the PMA effect separately at each time point. PMA treatment significantly affects TB at all incubation time points, while TM showed significant differences at 48 h. For ENT, a significant difference was observed at 48 h due to PMA treatment. ENT consumes bacteria for their protein synthesis and breaks down starch for survival (51, 52). As mentioned earlier, at 48 h, substrate depletion may have affected ENT survival, and PMA treatment could have effectively excluded nonviable ENT, decreasing its absolute abundance. MBB abundance decreased at 48 h due to the PMA treatment. As previously noted, methanogens derive energy from H_2_, but both TB and TP, which generate H_2_, decreased at 48 h. This suggests that the decrease in MBB abundance at 48 h was due to reduced H_2_ production, which limited substrate availability and thereby reduced the viability of MBB. The decrease in abundance may also result from PMA treatment, which excluded nonviable MBB. Consequently, the reduced abundance of methanogens likely led to a corresponding decrease in the associated methyl coenzyme-M reductase gene (*mcrA*).

### Effect of PMA treatment and incubation time on microbial composition and alpha diversity of ruminal microbiota

For bacteriota, PMA treatment effectively excluded bacteria presumed to be nonviable and significantly decreased the Shannon index, with no significant variation observed across incubation times. This reduction in diversity was primarily attributed to decreased evenness as the removal of nonviable bacteria may have allowed certain taxa to dominate the community. In contrast, incubation time alone did not lead to significant changes in the Shannon index, possibly due to a relatively stable balance between evenness and species richness throughout the fermentation period. Evenness and Simpson indices were also significantly reduced by PMA treatment, with both indices reaching their lowest values at 24 h. This reduction may be associated with the increased dominance of fast-growing, carbohydrate-degrading genera such as *Xylanibacter* and *Ruminobacter* at 24 h.

For archaeota, the absolute abundance increased at 48 h, supporting the observed rise in alpha diversity indices. Although the observed ASV richness increased over time, the overall archaeal ASV count was lower than that reported in a previous study (53), likely due to the limited taxonomic resolution of the V3–V4 region. Therefore, it may be inaccurate to attribute the increase in diversity indices directly to the effect of PMA treatment alone. Further studies employing higher-resolution sequencing are warranted to clarify these patterns.

Among the bacteriota phylum, Mycoplasmatota increased at all incubation time points after PMA treatment. Since *Anaeroplasma*, a member of this phylum, increased at all incubation time points after PMA treatment, the increase in Mycoplasmatota could be attributed to the proliferation of *Anaeroplasma. Anaeroplasma* lacks a cell wall, breaks down starch for energy, and requires lipopolysaccharide (LPS) for growth (54). The increase in *Anaeroplasma* at 0 h and 48 h, even under starch limitation, may result from its ability to utilize LPS from dead gram-negative bacteria or the inoculum, supporting its survival. At the genus level, PMA treatment at 48 h significantly reduced *Succinivibrio*, *Pseudoruminococcus*, *Ruminobacter*, and *Xylanibacter* abundance. These bacterial genera utilize easily degradable substrates such as starch. *Succinivibrio*, enriched in starch-fed rumen (55, 56), ferments glucose into acetate and succinate (57). *Pseudoruminococcus*, initially isolated from the human gut microbiota (58), degrades starch *in vitro* and produces butyrate (59). *Ruminobacter* ferments starch and maltose, yielding formic, acetic, and succinic acids (42, 60, 61). *Xylanibacter*, previously classified under *Prevotella* (62), ferments sugars such as cellobiose, glucose, and fructose, producing acetic and succinic acids (60). In the presence of vitamin B12, it shifts to propionic acid production (63). The observed reductions likely indicate substrate depletion over 48 h, compromising their survival and preventing amplification under PMA treatment. In contrast, *Succiniclasticum* showed an increase in differential abundance after PMA treatment at 48 h (LFC = 0.43). As a secondary utilizer of feed, it generates energy by converting succinate, produced by other microorganisms in the rumen, into propionate (64), likely supporting its survival and explaining the observed increase in its differential abundance.

At the genus level, significant changes in archaeota due to PMA treatment were observed only at 48 h, increasing the abundance of *Methanobrevibacter* and *Methanomicrobium*. Hydrogenotrophic methanogens, such as *Methanobrevibacter*, likely survived better during the extended incubation by utilizing H_2_ produced by other microorganisms, leading to their differential enrichment. These findings align with the decrease in methylotrophic Candidatus Thermoplasmatota and the increase in hydrogenotrophic Euryarchaeota at 48 h. However, microbial absolute abundance measurement revealed a decrease in *Methanobrevibacter*, likely due to a greater decline in the methylotrophic methanogen *Methanomassiliicoccus* (65, 66). *Methanomassiliicoccus* relies on methyl groups from amino acids such as methionine, which are abundant in protein-rich concentrate feeds (67). The depletion of concentrates at 48 h likely reduced methyl group availability, affecting *Methanomassiliicoccus* more severely. In contrast, hydrogenotrophic methanogens utilize H₂ produced by other microbes, enhancing their survival over *Methanomassiliicoccus*. These shifts highlight potential biases in traditional DNA-based analyses, underscoring the need for viability-PCR in microbiome studies.

### Effect of PMA treatment on the abundance of predicted enzyme commission numbers in relation to microbial metabolism

PMA treatment altered predicted functions, with these changes becoming more pronounced over time. In the bacterial functional profiles, enzymes such as alpha-amylase [EC:3.2.1.1], alpha-glucosidase [EC:3.2.1.20], and cellulase [EC:3.2.1.4], which break down the feed provided during incubation, showed decreased abundance after PMA treatment at 24 h. This suggests that some of the bacteria that break down fiber or starch to obtain energy may become nonviable as the available substrates deplete during the 24 h incubation. By 48 h, further substrate depletion amplified the differences caused by PMA treatment. Additionally, the increased abundance of ECs such as pyruvate dehydrogenase (acetyl-transferring) [EC:1.2.4.1], alcohol dehydrogenase [EC:1.1.1.1], and methylmalonyl-CoA mutase [EC:5.4.99.2] suggests that by 48 h, microorganisms utilizing the remaining substrates survived and contributed to VFA production. For example, the increased differential abundance of *Succiniclasticum,* which converts succinate to propionate, may have contributed to the upregulation of methylmalonyl-CoA mutase [EC:5.4.99.2] and succinate-CoA ligase (ADP-forming) [EC:6.2.1.5] (64). Additionally, the increase in ECs involved in lipopolysaccharide biosynthesis at 48 h, such as UDP-3-O-acyl-N-acetylglucosamine deacetylase [EC:3.5.1.108] and Kdo (2)-lipid IV(A) lauroyltransferase [EC:2.3.1.241], may result from the decrease in abundance of gram-positive bacteria such as *Butyrivibrio* and *Ruminococcoides*. A similar pattern was observed at the phylum level, with the abundance of Bacillota, which includes these genera, also decreasing. Bacillota primarily consists of gram-positive bacteria that degrade fibrous materials. *Butyrivibrio* utilizes cellulose, cellobiose, glucose, xylan, pectin, and arabinose as substrates (60), while *Ruminococcoides* specializes in starch degradation (68). At 24 h, no significant difference was observed in lipopolysaccharide-related functions, likely because sufficient fibrous substrates remained, supporting bacterial survival. However, by 48 h, substrate depletion likely reduced the viability of certain bacterial populations, and PMA treatment further excluded these nonviable cells. Since lipopolysaccharide is a component of the outer membrane in gram-negative bacteria (69), the decrease in the two gram-positive species, *Butyrivibrio* and *Ruminococcoides,* may account for the increased abundance of ECs involved in its biosynthesis. This result could also be influenced by the presence of *Anaeroplasma*. As an LPS-dependent species (54), *Anaeroplasma* may upregulate LPS-related metabolic pathways in response to starch depletion, potentially enhancing its survival under these conditions. However, these findings remain speculative, requiring further research on *Anaeroplasma* to confirm this hypothesis.

In archaeal functional profiles, PMA treatment likely increased the differential abundance of predominant hydrogenotrophic methanogens, such as *Methanobrevibacter*, leading to higher levels of coenzyme-B sulfoethylthiotransferase [EC:2.8.4.1], a key enzyme in methanogenesis (70). The increase in *Methanobrevibacter* and *Methanomicrobiumme* could also explain the observed increase in CoB—CoM heterodisulfide reductase [EC:1.8.98.1], an enzyme crucial for methanogen survival (71). This enzyme catalyzes the reduction of heterodisulfides to generate key substrates that enable the production of energy necessary for methanogen survival under anaerobic conditions.

### Exploring the potential of PMA treatment as an alternative to RNA-based analysis in rumen microbiome studies

This study showed the use of PMA to overcome the limitations of traditional DNA-based approaches (which involve nonviable microbes and make accurate analysis difficult), which suppresses DNA amplification from nonviable microbes. To the best of our knowledge, this is the first application of PMA in an *in vitro* rumen fermentation experiment and within a complex rumen microbiome. The limitations of RNA-based analyses (sampling time, RNA preservation, extraction efficiency, PCR detection limit, amongst others) often lead researchers to choose DNA-based approaches. Our findings indicate that PMA treatment in rumen samples excludes the amplification of DNA from microbes presumed to be nonviable, allowing more refined analysis than traditional DNA-based methods. Studies show that RNA-based analyses reveal higher abundance of Succinivibrionaceae family than those of the Prevotellaceae family, while DNA-based analyses often reveal a higher dominance of Prevotellaceae (10, 72, 73). In this experiment, PMA treatment increased *Ruminobacter* (LFC = 0.52) and *Succinivibrio* (LFC = 0.68), species of Succinivibrionaceae, at 0 h, further increase in *Ruminobacter* (LFC = 0.83) at 24 h, and decreased *Xylanibacter* (LFC = −0.39), a species of Prevotellaceae, at the same time point. These changes suggest that PMA treatment improves DNA-based analyses, aligning them more closely with RNA-based results. However, this trend is less pronounced at 48 h, likely due to substrate depletion over the incubation period, affecting both the microbial community structure and its activity. These findings align with patterns observed in Pseudomonadota, further supporting the trend observed at the genus level. Although PMA treatment improves DNA-based microbial analysis by targeting viable microorganisms, further validation is needed to confirm its comparability to RNA-based methods. In addition, as this study was based on pooled rumen fluid and conducted with technical replicates only, future studies incorporating biological replication will be essential to confirm the generalizability and robustness of these findings. Nevertheless, PMA has the potential to serve as a cost-effective and convenient alternative, providing a presumably active representation of the active microbial community compared to traditional DNA-based analysis.

## MATERIALS AND METHODS

Rumen contents were collected from five Hanwoo cows at a Nonghyup Co., Ltd. research farm using stomach tubing before their morning feeding. The samples were immediately pooled to generate a single inoculum, which was used in all experiments.

### Experiment 1: application of PMA treatment in rumen samples

The obtained rumen fluid was transferred into a thermos flask flushed with 99.999% CO_2_ gas and transported to the laboratory within 30 min. The fluid was filtered through two layers of cheesecloth, mixed with CO_2_-flushed *in vitro* buffer (74) at a 1:2 ratio, and bubbled with CO_2_ gas to maintain anaerobic conditions. A 50 mL aliquot of the resulting inoculum was transferred into a 125 mL serum bottle containing 0.25 g each of forage and concentrate (sieved through a 1 mm mesh), sealed with blue butyl rubber stoppers and aluminum caps to preserve anaerobic conditions, and incubated in a shaking incubator at 39°C and 60 rpm for 24 h. The *in vitro* substrate was composed of the same diet fed to donor animals, consisting of oat hay and a pelleted concentrate. For the fermentation experiment, a 1:1 ratio of forage to concentrate was used, which is commonly applied in *in vitro* studies to maintain experimental consistency. The detailed nutrient composition of these forages and concentrates is presented in Table S4. All donor cows had been fed this diet for at least 3 weeks before rumen fluid collection, ensuring microbial adaptation to the substrate. To support microbial growth, gas produced during anaerobic fermentation was removed with a syringe at 3, 6, and 12 h. After 24 h of incubation, the bottle was transferred to an anaerobic chamber (Whitley DG250 Anaerobic Workstation, Don Whitley Scientific, UK), where undiluted, fivefold, and tenfold diluted samples were prepared using an anaerobic dilution solution (ADS) modified from previous studies (75, 76). To confirm the proper function of PMA, a portion of each dilution was incubated in a water bath at 80°C for 20 min to induce damage in rumen microorganisms. For each dilution ratio (undiluted, fivefold, and tenfold), 12 experimental groups were established by combining two viability conditions (viable and nonviable) with or without PMA treatment. The PMA application experiment followed the procedure described in Section “PMA treatment.” Each experimental treatment was conducted using three technical replicates.

### Experiment 2: application of PMA treatment in *in vitro* digestibility experiment

Inoculum preparation for the *in vitro* digestibility experiment was identical to that used in Experiment 1. Samples were collected at three time points: immediately after inoculating the feed-containing bottles (0 h), after 24 h of incubation (24 h), and after 48 h of incubation (48 h). For each time point and replicate, tDNA, vDNA, and RNA were extracted from the same fermentation bottle to ensure that comparisons among sample types were not confounded by inter-bottle variation. Total gas production, methane production, pH, ammonia nitrogen (NH_3_-N), volatile fatty acids (VFA), dry matter digestibility (DMD), and neutral detergent fiber digestibility (NDFD) were analyzed using previously described methods (77). Total gas was collected in a gas bag with a pressure transducer (L20000DCV3, Laurel Electronics, USA), and methane concentration was determined by gas chromatography (YL6500 GC system, Youngin Chromass, Korea) using a packed GC column. pH was measured with a pH meter (MW150, Milwaukee Instruments, USA). NH_3_-N was measured using the colorimetric method described by Chaney and Marbach (78). VFA concentration was analyzed via gas chromatography (7890B GC system, Agilent Technologies, USA) with a Nukol-fused silica capillary column. DMD was determined using nylon bags (R510, Ankom Technology, USA). NDFD and acid detergent fiber digestibility (ADFD) were measured following the Ankom procedure with a fiber analyzer (A200, Ankom Technology, Macedon, NY, USA). DMD, NDFD, and ADFD calculations were performed according to the method described by Wei et al. (79). Each experimental treatment was conducted using five technical replicates.

### PMA treatment

The PMA treatment procedure was identical in Experiments 1 and 2. PMAxx (20 mM in H_2_O, 100 µL; Biotium, USA) was added to each sample to achieve a final concentration of 100 µM (39). All samples were prepared in autoclaved 2 mL screw-cap tubes (polypropylene, PP; Sarstedt, Germany). The tubes were covered with aluminum foil to create a dark environment, and PMA-treated samples were incubated at 25°C for 30 min, with inversion every 10 min. The samples were then exposed to light for 20 min using a PMA-Lite 2.0 LED Photolysis Device (Biotium, USA). All procedures were performed inside an anaerobic chamber. For samples without PMA treatment, the same procedure was followed, substituting ADS for PMA. Finally, the samples were centrifuged at 10,000 × *g* for 15 min at 4°C to remove the supernatant, leaving only the pellet, which was stored at −80°C.

### DNA extraction for 16S rRNA gene amplicon sequencing and quantitative real-time PCR (qPCR)

Total microbial DNA was extracted from the collected pellets of control and PMA-treated samples using the repetitive bead beating plus column purification (RBB + C) method described by (80). The concentration of the purified DNA was measured using a NanoDrop One microvolume UV-Vis spectrophotometer (Thermo Fisher Scientific, Wilmington, NC, USA) and immediately stored at −80°C.

The primer sets used for quantifying specific microbial populations were selected from previous research (Table S5). In Experiment 1, qPCR was conducted targeting total bacteria, protozoa, and methanogens. In Experiment 2, the same targets were analyzed, along with additional ones: total fungi, the methyl coenzyme-M reductase gene, *Entodinium* spp., *Dasytricha ruminantium, Methanobrevibacter* spp., and *Methanomicrobium* spp. The specificity of each primer set was validated using TestPrime (https://www.arb-silva.de/search/testprime/) (81). To generate qPCR standards, conventional PCR was performed on sample-derived DNA (82) using a PCR thermal cycler (TP 600, TaKaRa, Kusatsu, Gunma, Japan), with 1 µL of genomic DNA per reaction. After amplification, PCR products were separated via 1.5% agarose gel electrophoresis, before being purified using the AccuPrep PCR/Gel Purification Kit (Bioneer, Daejeon, Republic of Korea). The concentration of the purified products was then measured, following the same method used for sample DNA, and stored at −20°C. Microbial quantification was performed using the QuantStudio 1 system (Thermo Fisher Scientific, Wilmington, NC, USA). Each qPCR reaction utilized a TaqMan probe for TB (83) and PowerUp SYBR Master Mix (2X) for other microbial targets, as previously described (77). Microbial copy numbers were calculated using the equation provided by Singh et al. (84).

### Microbiome analysis

The V3–V4 regions of the 16S rRNA gene were amplified to construct sequencing libraries for each DNA sample. Sequencing was performed on the MiSeq platform (Illumina, San Diego, CA, USA). Microbial analysis was performed using QIIME2 (85). Primers were trimmed using Cutadapt (86). Quality filtering and chimeric sequence removal were performed with the DADA2 plugin (87). Bacteria and archaea were classified using RESCRIPt (88) by retrieving 16S rRNA reference data from the NCBI database (accessed on 20 June 2024). Further taxonomic filtering was used to remove unassigned sequences, chloroplasts, and mitochondria from the amplicon sequence variants (ASVs). Alpha diversity was assessed using several metrics, including richness (observed ASVs and Chao1 estimates), evenness, Faith’s phylogenetic diversity (Faith’s PD), Shannon’s index, and Simpson’s index. These metrics were computed from ASV tables averaged over 1,000 random sampling iterations at a sequence depth of 39,726 for bacteria and 77 for archaea (89).

Microbial metabolic functions were predicted from the ASVs using Phylogenetic Investigation of Communities by Reconstruction of Unobserved States 2 (PICRUSt2) (90). The coefficients of predicted enzyme commission (EC) abundance data were used to identify functional differences between PMA treatments across incubation time points. Additionally, overall comparisons of bacteriota and archaeota, based on normalized ASV and EC abundance data, were visualized using non-metric multidimensional scaling (NMDS) with a Bray-Curtis dissimilarity matrix. Visualizations were created using the ggplot2 package in R (ver. 4.4.0) (91).

### Statistical analysis

Levene’s test was used to assess the homogeneity of variance for fermentation data, alpha diversity indices, and microbial copy numbers from qPCR, while the Shapiro–Wilk test was used to evaluate normality. If the assumptions of normality or homoscedasticity were not met, the data were transformed using the Aligned Rank Transform (ARTool) (92) before statistical analysis. Statistical analyses were performed using PROC GLIMMIX in SAS 9.4 (SAS Institute Inc., Cary, NC, USA), with Tukey’s HSD test applied for *post hoc* comparisons. The Kruskal–Wallis test was used for single-factor analyses. Beta diversity indices and variations in microbial functional community profiles were assessed using PERMANOVA with Bray-Curtis dissimilarity in PAST software (version 4.03) (93). Bonferroni correction was applied to adjust for multiple comparisons. Differential microbial taxa and functional features were identified using ANCOM-BC (94).

In this study, major bacterial and archaeal taxa and functions present in at least 60% of the samples in at least one treatment group were analyzed. Taxa and functional features were included if they had an absolute Log-Fold Change (LFC) > 0.1 and an average relative abundance >0.5% (or 0.1% for bacterial functions). Statistical significance was determined using a *Q-*value threshold for microbial analyses and a *P*-value of <0.05 for all other datasets.

## Data Availability

The raw sequencing data generated in this study have been deposited in the NCBI Sequence Read Archive and are accessible through the BioProject accession number PRJNA1213268.

## SUPPLEMENTAL MATERIAL

Supplemental material aem.01429-25-s0001.docx Tables S1 to S5 and Fig. S1.

ASM does not own the copyrights to Supplemental Material that may be linked to, or accessed through, an article. The authors have granted ASM a non-exclusive, world-wide license to publish the Supplemental Material files. Please contact the corresponding author directly for reuse.
