# Supplementary material for: Refining microbial biomarker identification in rumen microbiome studies: a viability PCR-based approach
Source: Appl Environ Microbiol. 2025 Sep 19;91(10):e01429-25. doi: 10.1128/aem.01429-25 (PMC12542770; doi:10.1128/aem.01429-25)
Supplement: Supplemental material — Tables S1 to S5 and Fig. S1. [file aem.01429-25-s0001.docx]

| **Table S1.** Fermentation parameters measured over 24 and 48 h incubation times in the *in vitro* fermentation experiment (Experiment 2) | | | | |
| --- | --- | --- | --- | --- |
| **Parameter** | **Incubation time** | | **Pooled SEM** | ***P*-value** |
|  | **24 h** | **48 h** |  |  |
| DMD (%) | 71.40^B^ | 77.82^A^ | 0.48 | 0.001 |
| NDFD (%) | 39.67^B^ | 52.34^A^ | 0.83 | 0.001 |
| ADFD (%) | 17.75^B^ | 32.49^A^ | 1.27 | 0.001 |
| pH | 6.21^A^ | 6.08^B^ | 0.02 | 0.001 |
| NH_3_-N (mg/dL) | 15.78^B^ | 25.68^A^ | 0.40 | 0.001 |
| Total Gas (mL) | 118.97^B^ | 140.87^A^ | 2.61 | 0.001 |
| CH_4_ (mL) | 6.78^B^ | 9.41^A^ | 0.28 | 0.001 |
| CH_4_ (mL/g dDM) | 19.01^B^ | 24.18^A^ | 0.76 | 0.001 |
| Total VFA (mM) | 34.82^B^ | 44.63^A^ | 1.45 | 0.001 |
| BCVFA (%) | 0.88^B^ | 2.07^A^ | 0.12 | 0.001 |
| Acetate (%) | 66.96^A^ | 65.36^B^ | 0.51 | 0.016 |
| Propionate (%) | 19.83 | 19.48 | 0.13 | 0.122 |
| iso-butyrate (%) | 0.09^B^ | 0.53^A^ | 0.04 | 0.001 |
| Butyrate (%) | 11.54 | 11.94 | 0.30 | 0.275 |
| iso-valerate (%) | 0.79^B^ | 1.54^A^ | 0.08 | 0.001 |
| Valerate (%) | 0.79^B^ | 1.16^A^ | 0.07 | 0.001 |
| A:P ratio | 3.38 | 3.36 | 0.04 | 0.924 |
| ^A-B^, Means (n = 5) within a row followed by different superscripts indicate significant differences among treatments (*P* < 0.05).  DMD, dry matter digestibility; NDFD neutral detergent fiber digestibility; ADFD, acid detergent fiber digestibility; NH_3_-N, ammonia nitrogen; CH_4_ (mL/g dDM), methane production per degraded gram of dry matter; VFA, volatile fatty acid; BCVFA, branched-chain volatile fatty acid; 24 h, 24 hours of incubation; 48 h, 48 hours of incubation; SEM, standard error of the mean. | | | | |

| **Table S2.** Differential abundance analysis of bacterial genera to identify the effects of PMA treatment across different incubation times | | | |
| --- | --- | --- | --- |
| **Incubation time** | **Genus** | **Log-fold change** | ***Q-*value** |
| 0 h | *Succinivibrio* | 0.68 | < 0.001 |
|  | *Anaerovibrio* | 0.61 | 0.012 |
|  | *Parabacteroides* | 0.60 | < 0.001 |
|  | *Anaeroplasma* | 0.59 | < 0.001 |
|  | *Selenomonas* | 0.59 | < 0.001 |
|  | *Ruminobacter* | 0.52 | < 0.001 |
|  | *Succiniclasticum* | 0.37 | < 0.001 |
|  | *Xylanibacter* | 0.33 | 0.005 |
|  | *Aristaeella* | 0.21 | 0.042 |
|  | *Pseudoruminococcus* | -0.18 | 0.029 |
|  | *Butyrivibrio* | -0.79 | < 0.001 |
|  | *Treponema* | -0.89 | < 0.001 |
|  | *Ruminococcus* | -0.98 | < 0.001 |
|  | *Pseudobutyrivibrio* | -1.16 | < 0.001 |
|  | *Fibrobacter* | -1.18 | < 0.001 |
| 24 h | *Ruminobacter* | 0.83 | < 0.001 |
|  | *Anaerovibrio* | 0.73 | < 0.001 |
|  | *Selenomonas* | 0.67 | < 0.001 |
|  | *Anaeroplasma* | 0.64 | < 0.001 |
|  | *Succiniclasticum* | 0.59 | < 0.001 |
|  | *Parabacteroides* | 0.58 | < 0.001 |
|  | *Endomicrobium* | 0.42 | < 0.001 |
|  | *Ruminococcoides* | 0.34 | < 0.001 |
|  | *Aristaeella* | 0.31 | 0.012 |
|  | *Pseudoruminococcus* | -0.18 | 0.020 |
|  | *Xylanibacter* | -0.39 | < 0.001 |
|  | *Ruminococcus* | -0.58 | < 0.001 |
| 48 h | *Anaerovibrio* | 1.11 | < 0.001 |
|  | *Victivallis* | 1.09 | < 0.001 |
|  | *Fontisphaera* | 0.95 | < 0.001 |
|  | *Desulfovibrio* | 0.67 | < 0.001 |
|  | *Oligosphaera* | 0.43 | < 0.001 |
|  | *Succiniclasticum* | 0.43 | < 0.001 |
|  | *Anaeroplasma* | 0.27 | < 0.001 |
|  | *Ruminococcoides* | -0.29 | < 0.001 |
|  | *Vescimonas* | -0.65 | < 0.001 |
|  | *Treponema* | -0.75 | < 0.001 |
|  | *Endomicrobium* | -1.16 | < 0.001 |
|  | *Butyrivibrio* | -1.59 | < 0.001 |
|  | *Xylanibacter* | -1.66 | < 0.001 |
|  | *Ruminobacter* | -2.54 | < 0.001 |
|  | *Pseudoruminococcus* | -2.97 | < 0.001 |
|  | *Succinivibrio* | -3.26 | < 0.001 |
| The table includes only major bacterial genera, found in more than 60% of the samples in at least one treatment group with an average relative abundance at least over 0.5% were presented, which significantly affected by PMA treatment (*Q* < 0.05), with an absolute log-fold change (LFC) threshold of > 0.1. Positive LFC values indicate higher abundance in the PMA treatment group, while negative LFC values indicate higher abundance in the DNA group. | | | |

| **Table S3.** Differential abundance analysis of archaeal genera at 48 h of incubation to identify the effects of PMA treatment | | | |
| --- | --- | --- | --- |
| **Incubation time** | **Genus** | **Log-fold change** | ***Q-*value** |
| 48 h | *Methanobrevibacter* | 0.62 | < 0.001 |
|  | *Methanomicrobium* | 0.68 | < 0.001 |
| The table includes only major archaeal genera, found in more than 60% of the samples in at least one treatment group with an average relative abundance at least over 0.5% were presented, which significantly affected by PMA treatment (*Q* < 0.05), with an absolute log-fold change (LFC) threshold of > 0.1. Positive LFC values indicate higher abundance in the PMA treatment group.  No significant PMA treatment effects were observed at 0 h and 24 h of incubation. | | | |

| **Table S4.** Nutritional composition (on a dry matter basis) of the oat hay and pelleted concentrate diet used both for feeding donor animals and as the substrate for *in vitro* fermentation in Experiments 1 and 2. | | |
| --- | --- | --- |
| **Items** | **Oat hay (%)** | **Pellet concentrate (%)** |
| Dry matter | 91.56 | 89.19 |
| Crude protein | 3.55 | 16.20 |
| Crude fat | 2.15 | 4.08 |
| Crude fiber | 27.40 | 8.66 |
| Crude ash | 5.02 | 6.95 |
| Calcium | 0.09 | 1.22 |
| Phosphorus | 0.09 | 0.65 |
| ADF | 30.87 | 13.23 |
| NDF | 54.17 | 31.10 |

| **Table S5.** Primer sets used in experiments 1 and 2 | | | | | | |
| --- | --- | --- | --- | --- | --- | --- |
| **Species or microbial group** | **Target gene** | **Primer name** | **Sequence (5'-3')** | **Amplicon size (bp)** | **PCR conditions** | **Reference** |
| *Entodinium* spp. | 18S rRNA gene | Oph-151F | GAGCTAATACATGCTAAGGC | 317 | 95°C (15 s) → 55°C (30 s) → 72°C (30 s) | (1) |
|  |  | Ento-472R | CCCTCACTACAATCGAGATTTAAGG |  |  |  |
| *Dasytricha ruminantium* | 18S rRNA gene | Iso-Das-151F | CTAGAGCTAATACATGCC | 312 | 95°C (15 s) → 55°C (30 s) → 72°C (30 s) | (1) |
|  |  | Das-472R | CTACAATCACAATTAAATTGC |  |  |  |
| *Methanobrevibacter* spp. | 16S rRNA gene | NestMbbF | TGGGAATTGCTGGWGATACTRTT | 231 | 95°C (30 s) → 60°C (30 s) → 72°C (30 s) | (2) |
|  |  | NestMbbR | GGAGCRGCTCAAAGCCA |  |  |  |
| *Methanomicrobium* spp. | 16S rRNA gene | NestMmF | TGTTTAAAACACATGGGAAGA | 176 | 95°C (30 s) → 60°C (30 s) → 72°C (30 s) | (2) |
|  |  | NestMmR | ATTCCCAGTATCTCTTAGACGC |  |  |  |
| Total bacteria | 16S rRNA gene | 340f | TCCTACGGGAGGCAGCAGT | 467 | 95°C (15 s) → 60°C (60 s) | (3) |
|  |  | 806r | GGACTACCAGGGTATCTAATCCTGTT |  |  |  |
| Total protozoa | 18S rRNA gene | 316f | GCTTTCGWTGGTAGTGTATT | 234 | 94°C (30 s) → 54°C (30 s) → 72°C (60 s) | (4) |
|  |  | 539r | CTTGCCCTCYAATCGTWCT |  |  |  |
| Total methanogen | 16S rRNA gene | Met86f | GCTCAGTAACACGTGG | 791 | 95°C (15 s) → 55°C (30 s) → 72°C (60 s) | (2) |
|  |  | Met915r | GTGCTCCCCCGCCAATTCCT |  |  |  |
|  | methyl coenzyme-M reductase gene | mcrA-f | GGTGGTGTMGGATTCACACARTAYGCWACAGC | 488 | 95°C (15 s) → 60°C (60 s) | (5) |
|  |  | mcrA-r | TTCATTGCRTAGTTWGGRTAGTT |  |  |  |
| Total fungi | ITS1 | Total Fungi-f | CTTGGTCATTTAGAGGAAGTA | 433 | 94°C (60 s) → 58°C (30 s) → 72°C (45 s) | (6) |
|  | 5.8S rRNA | Total Fungi-r | GTGCAATATGCGTTCGAAGATT |  |  |  |


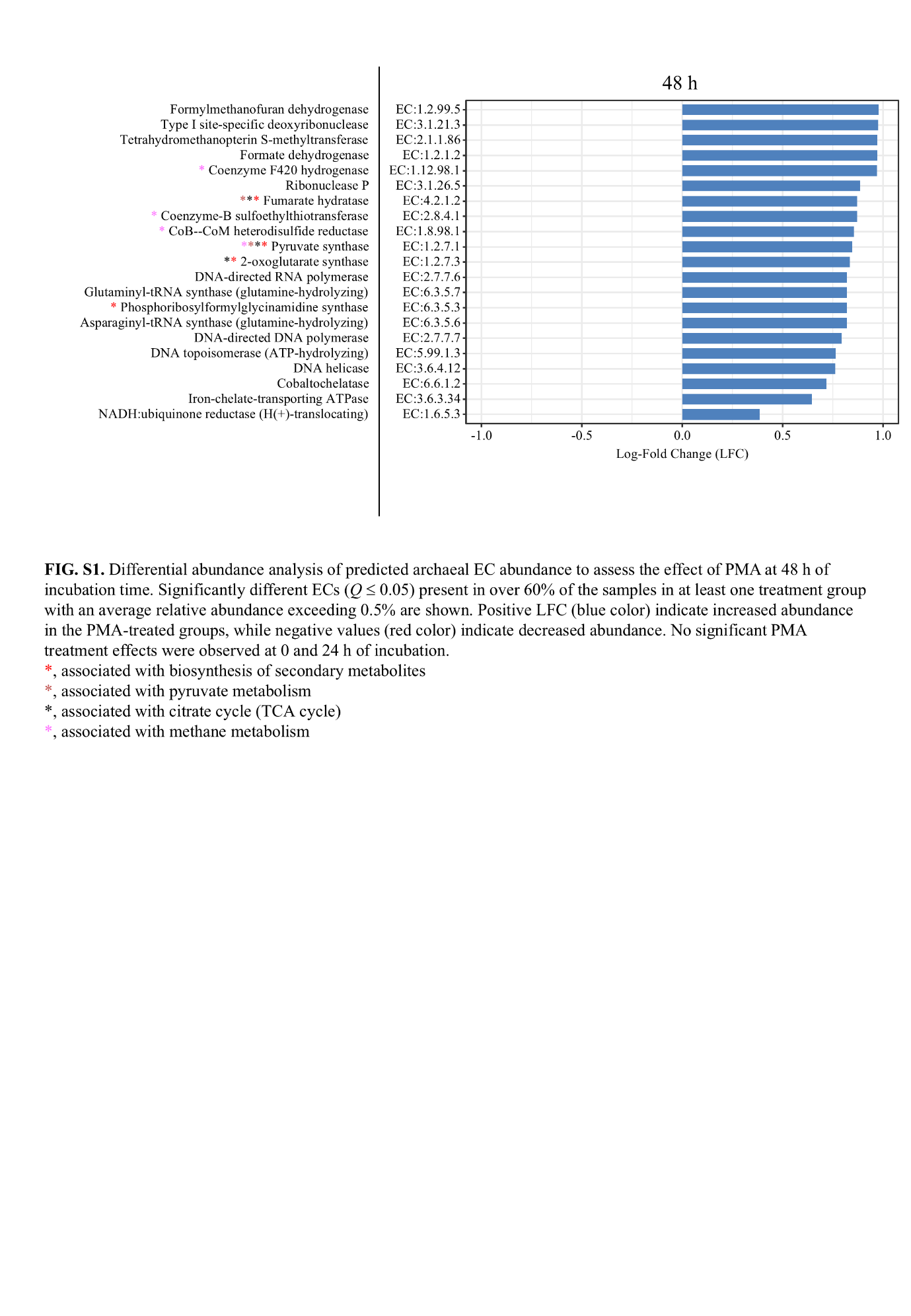


**References**

1. Skillman LC, Toovey AF, Williams AJ, Wright AD. 2006. Development and validation of a real-time PCR method to quantify rumen protozoa and examination of variability between *Entodinium* populations in sheep offered a hay-based diet. Appl Environ Microbiol 72:200–206.

2. Tymensen LD, McAllister TA. 2012. Community structure analysis of methanogens associated with rumen protozoa reveals bias in universal archaeal primers. Appl Environ Microbiol 78:4051–4056.

3. Stiverson J, Morrison M, Yu Z. 2011. Populations of select cultured and uncultured bacteria in the rumen of sheep and the effect of diets and ruminal fractions. Int J Microbiol 2011:750613.

4. Sylvester JT, Karnati SK, Yu Z, Morrison M, Firkins JL. 2004. Development of an assay to quantify rumen ciliate protozoal biomass in cows using real-time PCR. J Nutr 134:3378–3384.

5. Luton PE, Wayne JM, Sharp RJ, Riley PW. 2002. The mcrA gene as an alternative to 16S rRNA in the phylogenetic analysis of methanogen populations in landfill. Microbiology (Reading) 148:3521–3530.

6. Fliegerova KO, Podmirseg SM, Vinzelj J, Grilli DJ, Kvasnová S, Schierová D, Sechovcová H, Mrázek J, Siddi G, Arenas GN, Moniello G. 2021. The effect of a high-grain diet on the rumen microbiome of goats with a special focus on anaerobic fungi. Microorganisms 9:157.
